# Supplementary material for: An injectable hydrogel enhances tissue repair after spinal cord injury by promoting extracellular matrix remodeling
Source: Nat Commun. 2017 Sep 14;8:533. doi: 10.1038/s41467-017-00583-8 (PMC5599609; doi:10.1038/s41467-017-00583-8)
Supplement: Supplementary file 1 — Supplementary Information [file 41467_2017_583_MOESM1_ESM.pdf]

Description of Supplementary Files

File name: Supplementary Information
Description: Supplementary figures.

File name: Supplementary Movie 1
Description: Three-dimensional reconstruction of the injury site in an animal with PBS injection. Spinal cord tissue from an animal with PBS injection was three-dimensionally reconstructed using Neurolucida software. The animal was sacrificed 4 weeks after PBS injection (5 weeks after injury).

File name: Supplementary Movie 2
Description: Three-dimensional reconstruction of the injury site in an animal with injection of I-5 hydrogel. Spinal cord tissue from an animal with I-5 injection was three-dimensionally reconstructed using Neurolucida software. The animal was sacrificed 4 weeks after injection (5 weeks after injury).

File name: Supplementary Movie 3
Description: Composite 3D movie of the spinal cord tissue in PBS group imaged with LSFM following tissue clearing. The entire spinal cord tissue block covering the lesion site was processed for tissue clearing as described in the methods section. Then, LSFM images were taken and the stacked images were made into a composite 3D movie. The cavity spaces were identified on the basis of the lack of autofluorescence signal and color-coded with magenta. Animals with PBS injection usually showed big and multiple cavities expanding rostrocaudally.

File name: Supplementary Movie 4
Description: Composite 3D movie of the spinal cord tissue in I-5 group imaged with LSFM following tissue clearing. The entire spinal cord tissue block covering the lesion site was processed for tissue clearing as described in the methods section. Then, LSFM images were taken and the stacked images were made into a composite 3D movie. The cavity spaces were identified on the basis of the lack of autofluorescence signal and color-coded with magenta. The size of cavity was consistently very small in I-5 group.

File name: Peer review file

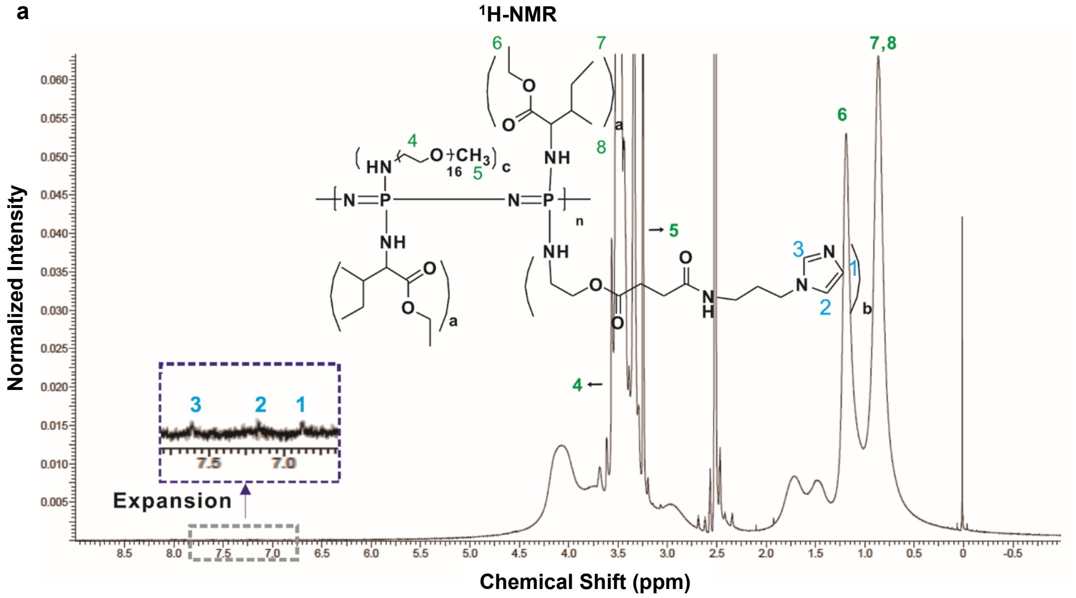

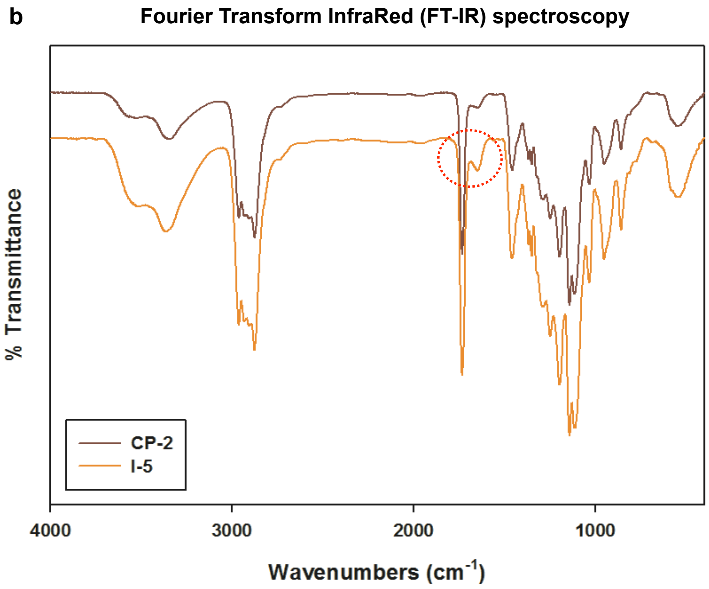

Supplementary Figure 1. Characterizations of I-5 hydrogel

(a) 1H-NMR analysis of I-5 hydrogel. The numbers above the individual peaks indicate the corresponding molecular structures with the same numbers. Specific peaks for the imidazole group, peaks 1 and 2, were observed at ppm 7 and 7.5, respectively. (b) Fourier Transform InfraRed (FT-IR) spectrum of CP-2 and I-5. Absorption by the amide bond between CP-2 and 1-3 aminopropylimidazole was increased in I-5 (red dotted circle).

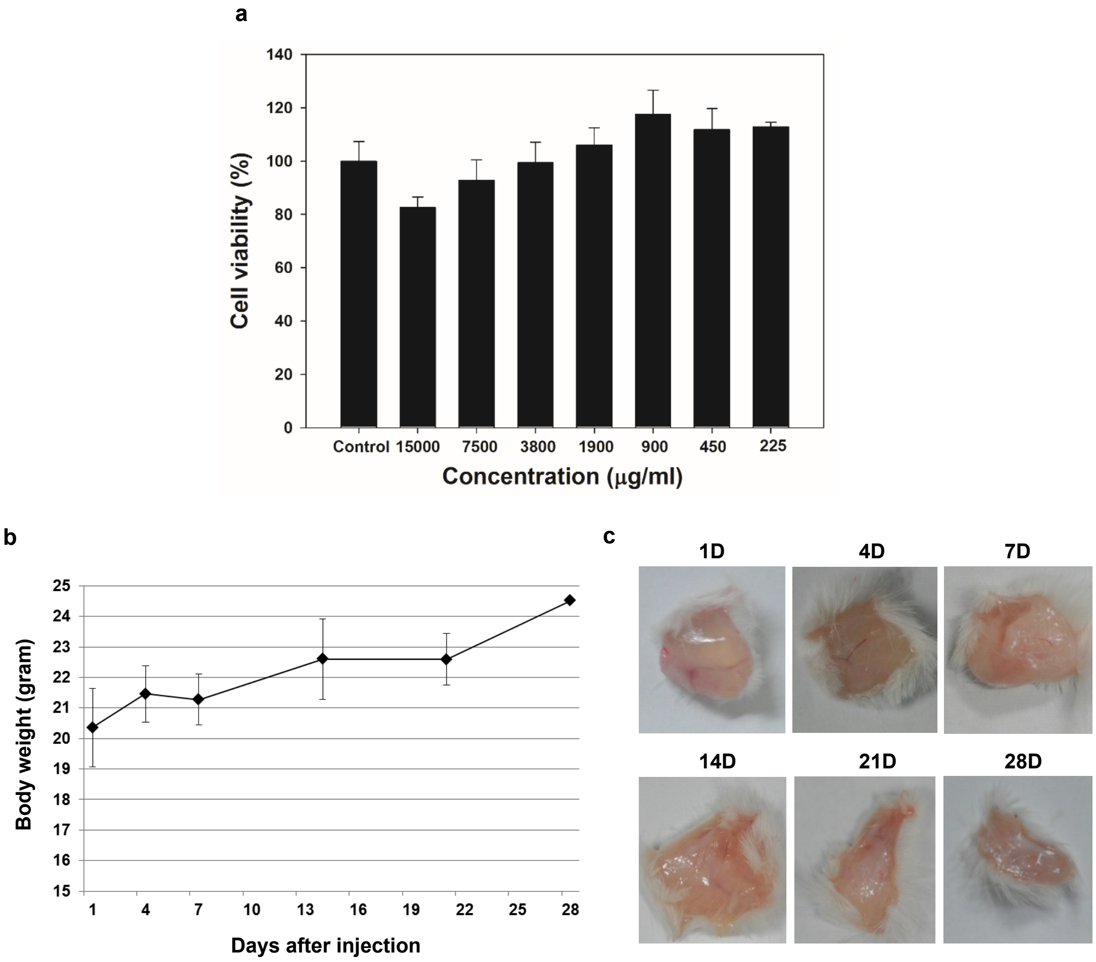

Supplementary Figure 2. Biocompatibility of I-5 hydrogel

(a) Assessment of cytotoxicity of I-5. The viability of cultured NIH3T3 cells exposed to varying concentrations of I-5 polymer was measured using the EZ-CYTOX assay kit. N = 5 or 6 replicates per concentration. Error bars represent standard deviation. (b) Measurement of body weight. After I-5 solution was injected into the dorsal subcutis, body weight was measured at designated time points up to 28 days (D, days). N = 6 animals. Error bars represent standard deviation. (c) Visual inspection of the injection sites did not reveal any evidence of inflammation or tissue damage in the subcutaneous tissue surrounding the hydrogel.

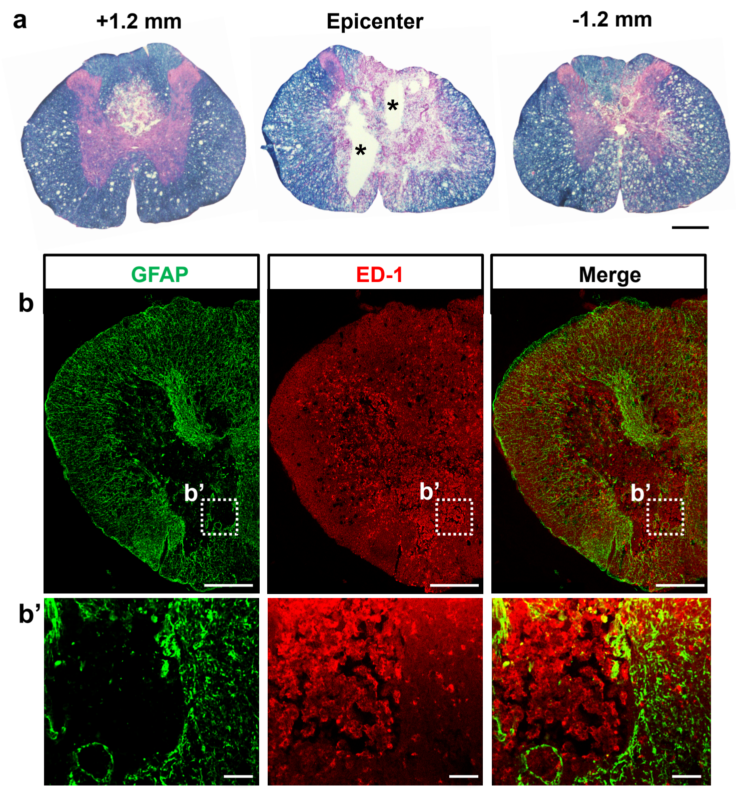

Supplementary Figure 3. Cystic cavities formed in the lesioned spinal cord 7 days after injury

(a) Formation of cystic cavities 7 days after contusion injury. Representative images of transverse spinal cord sections stained with eriochrome cyanine and eosin. The sections shown are from the epicenter and 1.2 mm rostral (+1.2 mm) o caudal (-1.2 mm) to it. Asterisks indicate cystic cavities. Scale bar represents 200 μm. (b) Cellular characteristics of the non-cystic lesions at the epicenter 7 days after injury. A transverse spinal cord section was stained with GFAP and CD68 antibodies. The lesion area was packed with CD68 positive macrophages surrounded by GFAP positive astrocytes. The boxed regions in (b) were magnified in (b'). Scale bars represent 200 μm in (b) and 50 μm in (b').

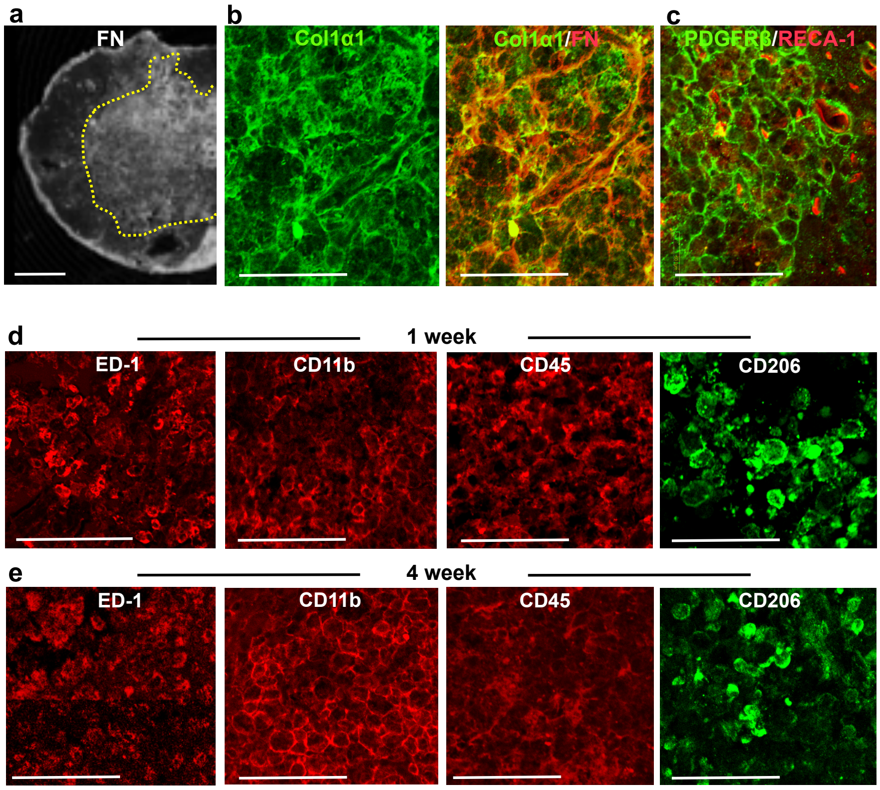

Supplementary Figure 4. Cellular composition in the FN-rich ECM induced by I-5

(a-c) Cellular sources for FN-rich ECM in animals with I-5 injection. (a) FN-rich ECM within which the following images were obtained. The yellow dotted line indicates the boundary of the FN-rich ECM. (b) Abundant Collagen 1α1 (Col1α1) immunoreactivity (green) was observed within the FN-rich ECM. The Col1α1 positive signal was colocalized with FN immunoreactivity (red). (c) PDGF receptor β (PDGFRB) immunoreactivity (green) encircling RECA-1 positive endothelial cells (red) was also frequently observed within the FN-rich ECM. (d, e) Presence of macrophages in the FN-rich ECM at 1 (d) and 4 weeks (e) after I-5 injection. Macrophages were positive with ED-1, CD11b, CD45, and CD206. Scale bar in (a) represents 200 μm. All the other scale bars represent 100 μm.

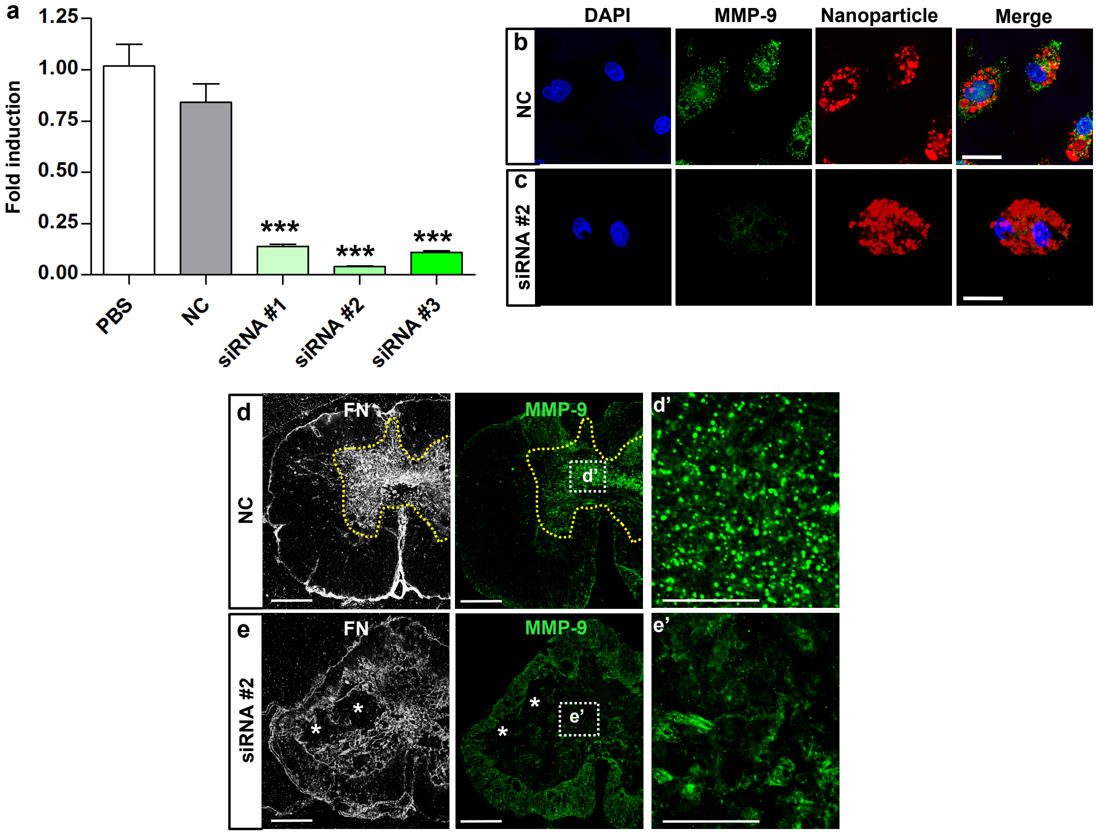

Supplementary Figure 5. Validation of MMP-9 knockdown by siRNA nanoparticles

(a) Measurement of mRNA levels by real-time PCR. Primary peritoneal macrophages were treated with three MMP-9 siRNAs with different sequences conjugated to nanoparticles. All three siRNAs effectively reduced the MMP-9 mRNA level compared to PBS or non-targeting control (NC) siRNA, with the #2 sequence having the largest effect. *** indicates $p < 0.001$ by one-way ANOVA followed by Tukey's *posthoc* analysis. Error bars represent the SEM. (b, c) Validation of MMP-9 knockdown at the protein level. Peritoneal macrophages were treated with either NC siRNA (b) or MMP-9 siRNA #2 (c). The nanoparticles labeled with red fluorescence were shown in red within macrophages. Scale bars represent 20 µm. (d, e) Knockdown of MMP-9 *in vivo*. Representative images of transverse spinal cord sections from animals injected with I-5 mixed with NC (d) or MMP-9 siRNA (e). The yellow dotted line indicates the boundary of the FN-rich matrix. In animals injected with I-5 mixed with MMP-9 siRNA, very little MMP-9 immunoreactivity was observed in the FN immunoreactive matrix surrounding cystic cavities indicated by asterisks. The boxed regions in (d, e) were magnified in (d', e'). All scale bars represent 100 µm.

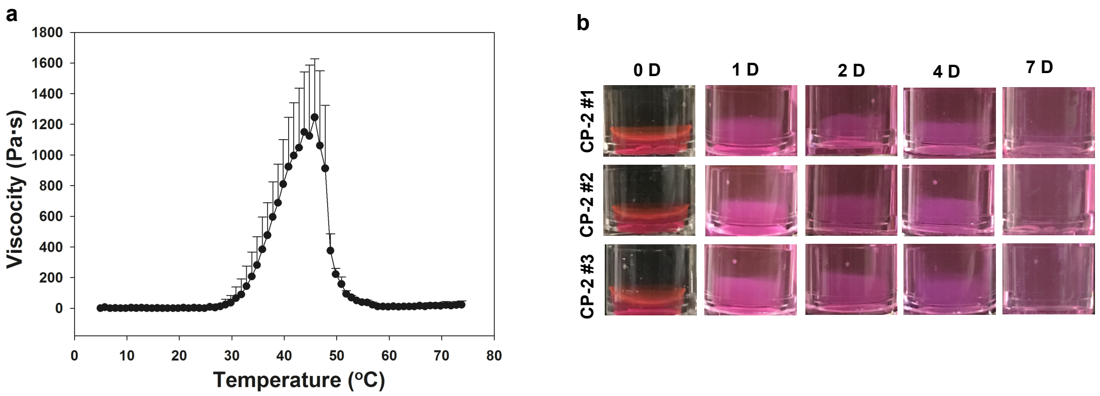

Supplementary Figure 6. Sol-gel transition properties and *in vitro* stability test of CP-2 hydrogel.

(a) Temperature-dependent changes in viscosity of CP-2 measured by a viscometer. Error bars represent standard deviation. (b) *In vitro* stability test. Three independent CP-2 gels were monitored until 7 days *in vitro* at 37°C.
